# Supplementary material for: The orphan ligand, activin C, signals through activin receptor-like kinase 7
Source: eLife. 2022 Jun 23;11:e78197. doi: 10.7554/eLife.78197 (PMC9224996; doi:10.7554/eLife.78197)
Supplement: Supplementary file 1. — The activity data corresponding to Figures 1B, D, 4C, 5E were imported into GraphPad prism and fit using a non-linear regression to calculate the EC50 or IC50 with standard error was calculated from triplicate experiments. [file elife-78197-supp1.docx]

Supplemental File 1 – EC50 and IC50 values for luciferase assays

| **Figure 1B:** | **EC50; Error (nM)** |
| --- | --- |
| ActA | 0.16; 0.02 |
| ActAC | 16; 11 |
| ActC | - |
| ActB | 0.13; 0.06 |
| **Figure 1D (ALK4):** |  |
| ActA | 0.76; 0.25 |
| ActAC | 0.90; 0.64 |
| ActC | - |
| ActB | 0.23; 0.10 |
| **Figure 1D (ALK7):** |  |
| ActA | - |
| ActAC | 0.14; 0.05 |
| ActC | 0.45; 0.13 |
| ActB | 0.1; 0.06 |
|  |  |
| **Figure 4C:** | **EC50; Error (nM)** |
| ActA | 0.15;.06 |
| ActC | 0.08; .03 |
|  |  |
| **Figure 5E:** | **EC50; Error (nM)** |
| ActC IQP | 1.7; 0.83 |
| ActC IAP | 4.1; 0.2 |
| ActC RnD | 1.2; 0.63 |
