# Supplementary material for: The orphan ligand, activin C, signals through activin receptor-like kinase 7
Source: eLife. 2022 Jun 23;11:e78197. doi: 10.7554/eLife.78197 (PMC9224996; doi:10.7554/eLife.78197)
Supplement: Supplementary file 2. [file elife-78197-supp2.docx]

Supplemental File 2 – Surface Plasmon Resonance

| **Analyte** | **Ligand** | **K_a_ (M^-1^s^-1^) x 10^6^** | **K_d_ (s^-1^) x 10^-4^** | **K_D_ (pM)^a^** |
| --- | --- | --- | --- | --- |
| Activin A  Activin A  Activin A  Activin A | ActRIIA-Fc  ActRIIB-Fc  ActRIIB-ALK4-Fc  ActRIIB-ALK7-Fc | 7.2 ± 1.4  7.7 ± 1.0  7.0 ± 0.77  2.0 ± 0.15 | 1.6 ± 0.33  0.60 ± 0.055  2.1 ± 0.20  8.9 ± 0.09 | 22 ± 0.030  8.1 ± 1.8  30 ± 6.2  310 ± 12 |
| Activin AC  Activin AC  Activin AC  Activin AC | ActRIIA-Fc  ActRIIB-Fc  ActRIIB-ALK4-Fc  ActRIIB-ALK7-Fc | 2.9 ± 0.040  2.4 ± 0.16  2.1 ± 0.34  5.6 ± 0.23 | 4.3 ± 0.39  2.1 ± 0.080  9.2 ± 0.26  2.8 ± 0.12 | 150 ± 12  90 ± 2.6  460 ± 88  51 ± 0.050 |
| Activin C  Activin C  Activin C | ActRIIA-Fc  ActRIIB-Fc  ActRIIB-ALK7-Fc | Transient Binding  Transient Binding  0.25 ± .0075 | Transient Binding  Transient Binding  5.7 ± 0.060 | -  -  2200 ± 44 |
| ActC IAP | ActRIIA-Fc | - | - | 620 ± 220^b^ |
| Activin B  Activin B  Activin B | ActRIIA-Fc  ActRIIB-Fc  ActRIIB-ALK7-Fc | 7.7 ± 0.15  7.2 ± 0.22  14 ± 0.0 | 0.73 ± 0.0020  2.1 ± 0.36  .58 ± 0.10 | 9.5 ± 0.21  30 ± 5.9  4.0 ± 0.70 |

^a^Kinetic parameters were analyzed using the Biacore T200 evaluation software using a 1:1 binding model and are the average of two independent, replicate experiments.

^b^Kinetic parameters were analyzed using the Biacore T200 evaluation software using a steady state analysis and are the average of two independent, replicate experiments.
