# Supplementary material for: The orphan ligand, activin C, signals through activin receptor-like kinase 7
Source: eLife. 2022 Jun 23;11:e78197. doi: 10.7554/eLife.78197 (PMC9224996; doi:10.7554/eLife.78197)
Supplement: Supplementary file 3. [file elife-78197-supp3.docx]

Supplemental File 3 – Primers used for RT-qPCR

| **Gene** | **Forward Primer Sequence** | **Reverse Primer Sequence** |
| --- | --- | --- |
| *Rp119* | 5’-CGGGAATCCAAGAAGATTGA-3’ | 5’-TTCAGCTTGTGGATGTGCTC-3’ |
| *Pparg2* | 5’-TTCGCTGATGCACTGCCTAT-3’ | 5’-GGAATGCGAGTGGTCTTCCA-3’ |
| *Pnpla2* | 5’-CTCACATCTACGGAGCCTCG-3’ | 5’-CGGATGGTCTTCACCAGGTT-3’ |
| *CEBPa* | 5’-TTCGGGTCGCTGGATCTCTA-3’ | 5’-TCAAGGAGAAACCACCACGG-3’ |
