# Supplementary figures and images for: The orphan ligand, activin C, signals through activin receptor-like kinase 7
Source: eLife. 2022 Jun 23;11:e78197. doi: 10.7554/eLife.78197 (PMC9224996; doi:10.7554/eLife.78197)

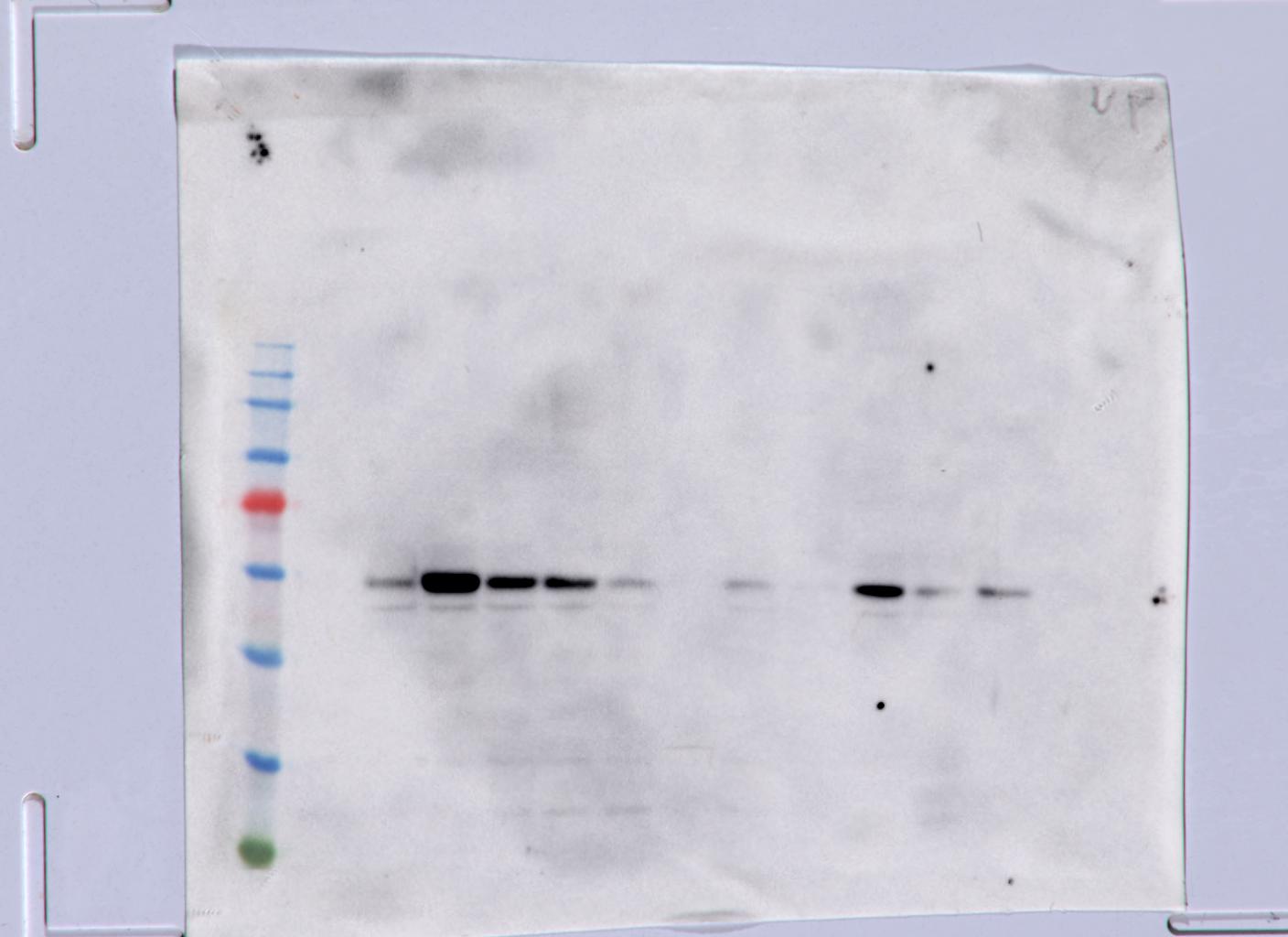

Supplement: Source data 1. [file elife-78197-data1.zip › ActC_Source_Files/ActC_SFig4_BottomLeft_pSmad2.jpg]

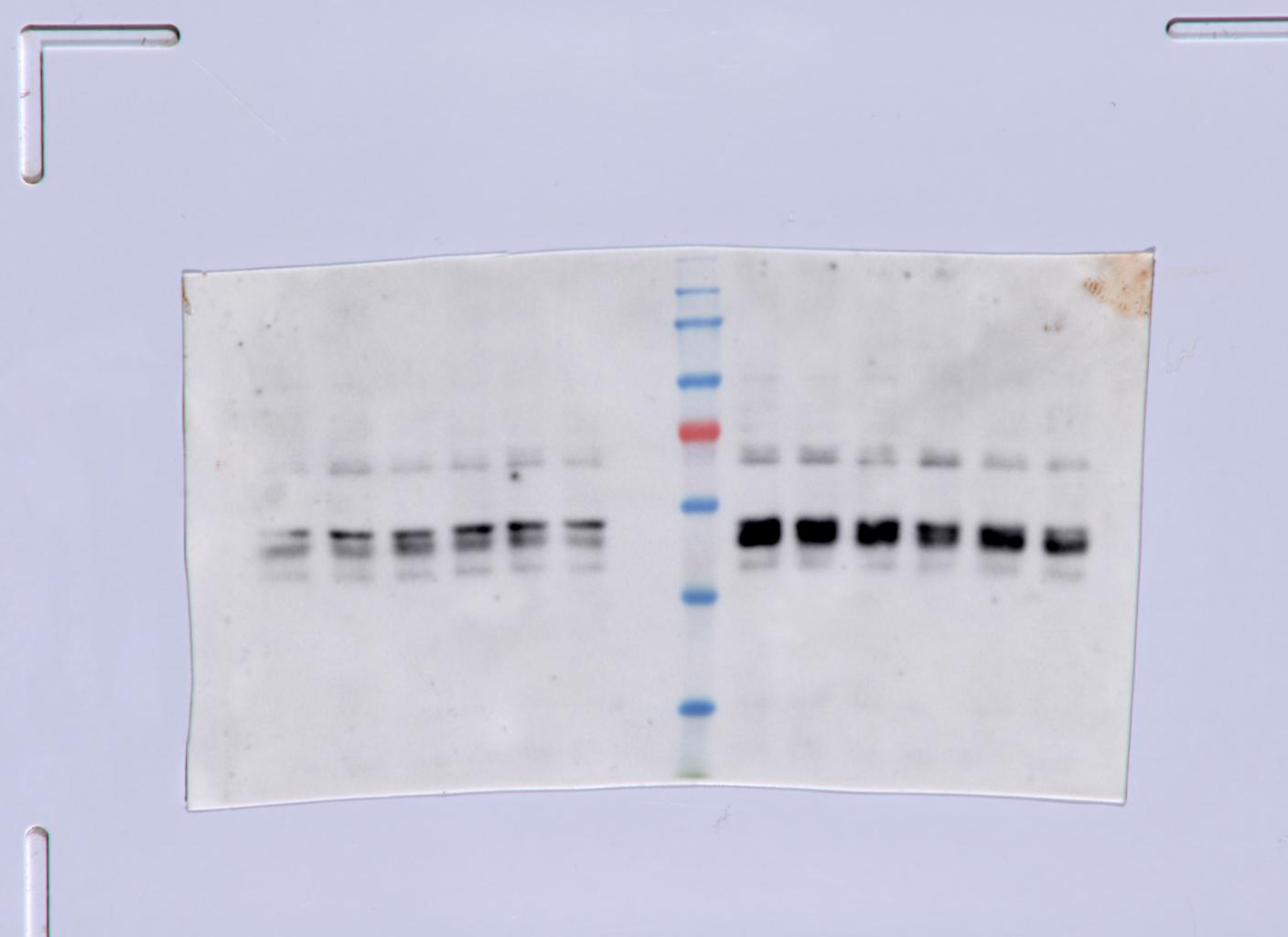

Supplement: Source data 1. [file elife-78197-data1.zip › ActC_Source_Files/ActC_SFig4_BottomMiddle_Smad2.jpg]

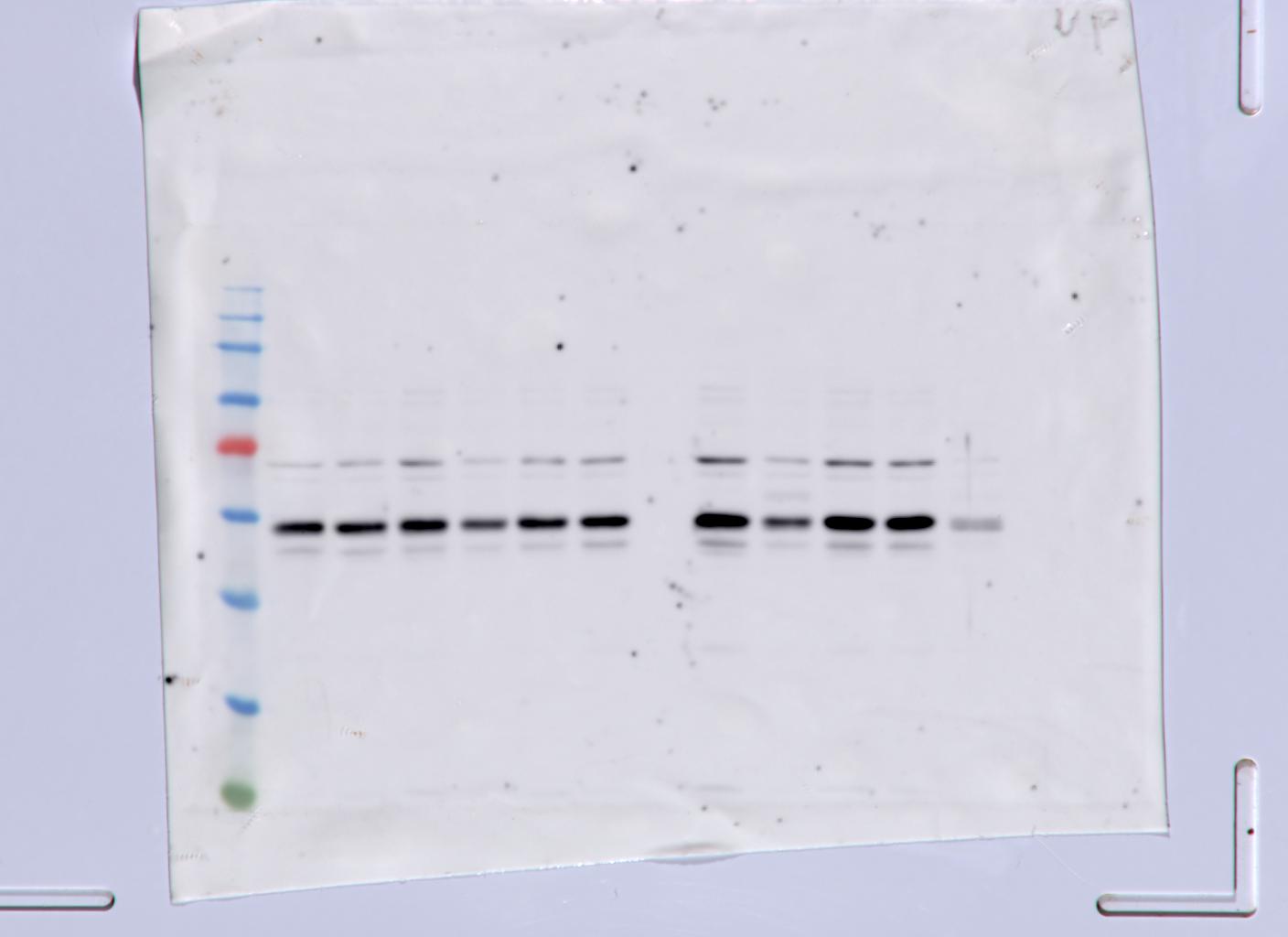

Supplement: Source data 1. [file elife-78197-data1.zip › ActC_Source_Files/ActC_SFig4_BottomLeft_Smad2.jpg]

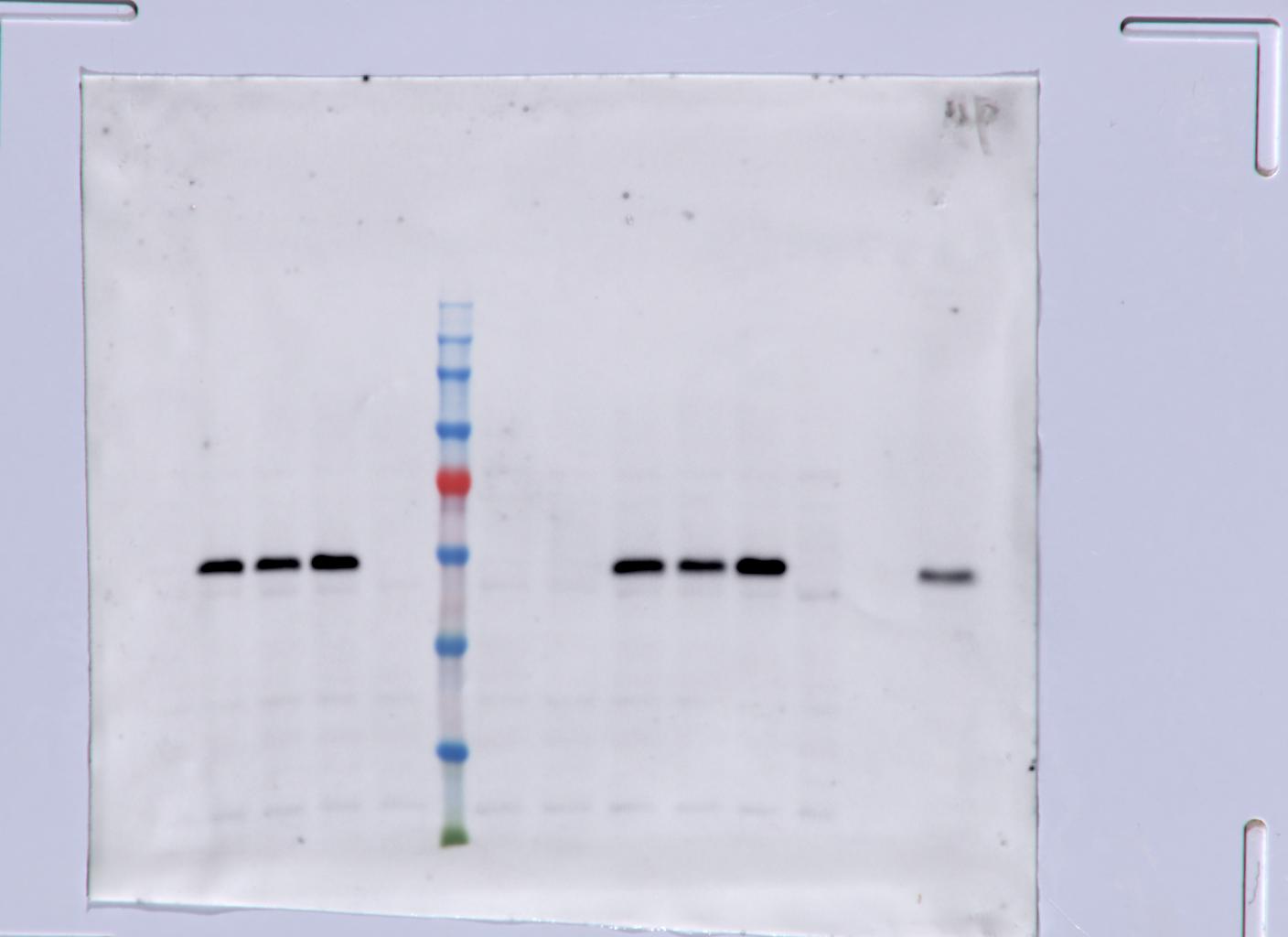

Supplement: Source data 1. [file elife-78197-data1.zip › ActC_Source_Files/ActC_SFig4_TopMiddle_pSmad2.jpg]

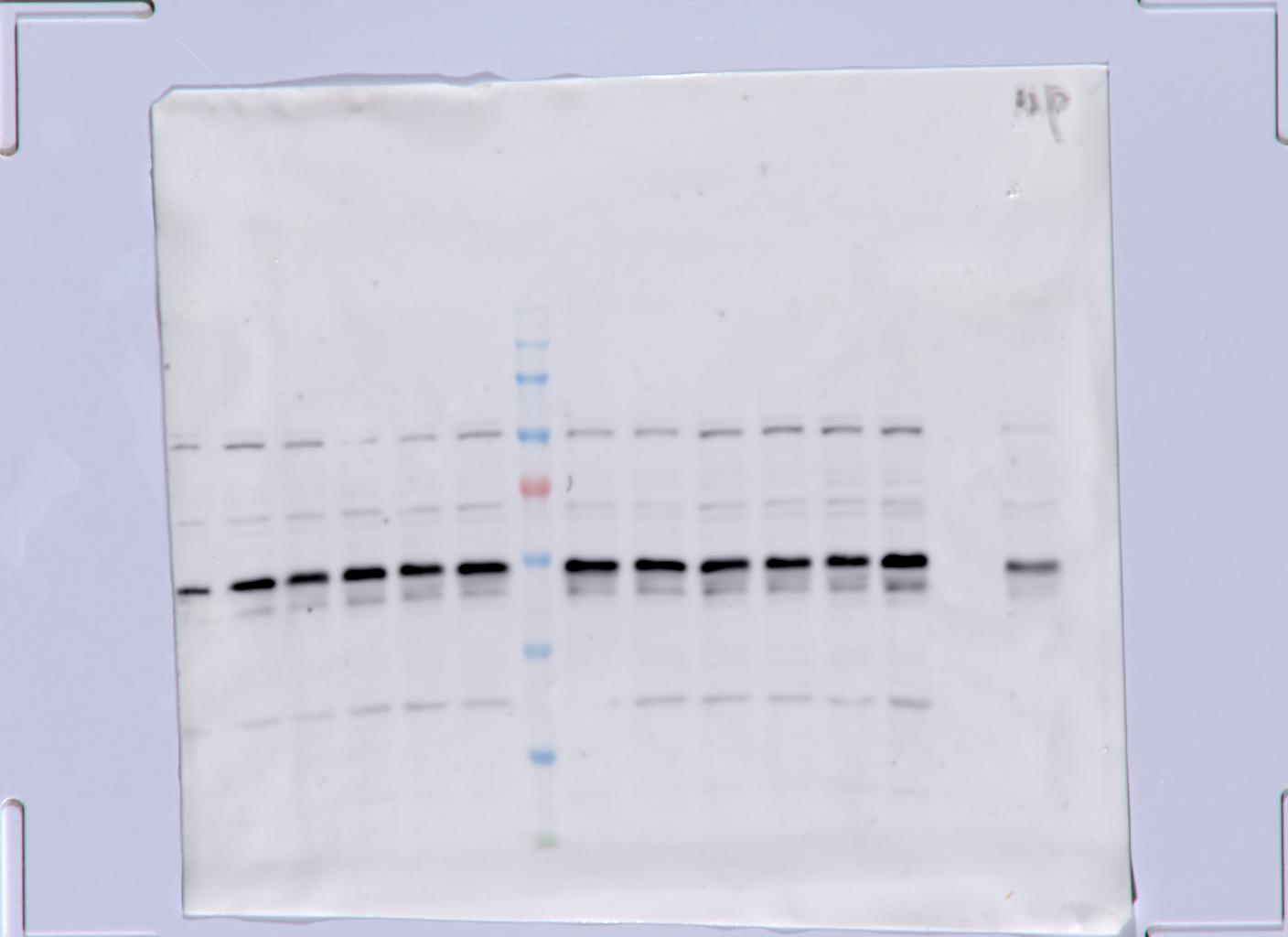

Supplement: Source data 1. [file elife-78197-data1.zip › ActC_Source_Files/ActC_SFig4_TopMiddle_Smad2.jpg]

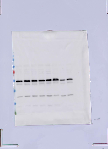

Supplement: Source data 1. [file elife-78197-data1.zip › ActC_Source_Files/ActC_SFig4_BottomRight_Smad2.png]

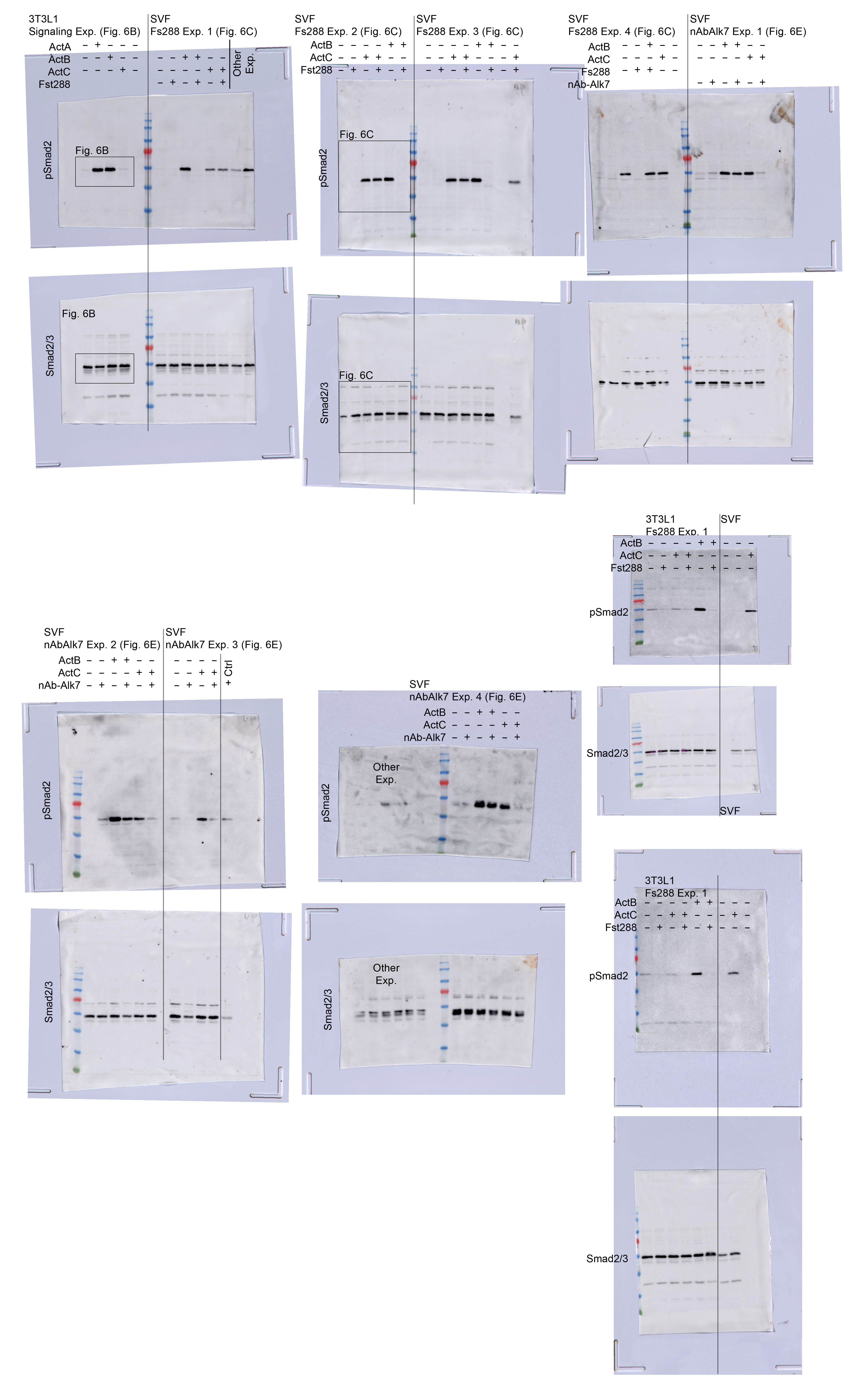

Supplement: Source data 1. [file elife-78197-data1.zip › ActC_Source_Files/ActC_Figure6_SourceFile2_Raw_westerns_Labeled.png]

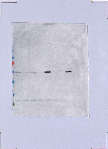

Supplement: Source data 1. [file elife-78197-data1.zip › ActC_Source_Files/ActC_SFig4_BottomRight_pSmad2.png]

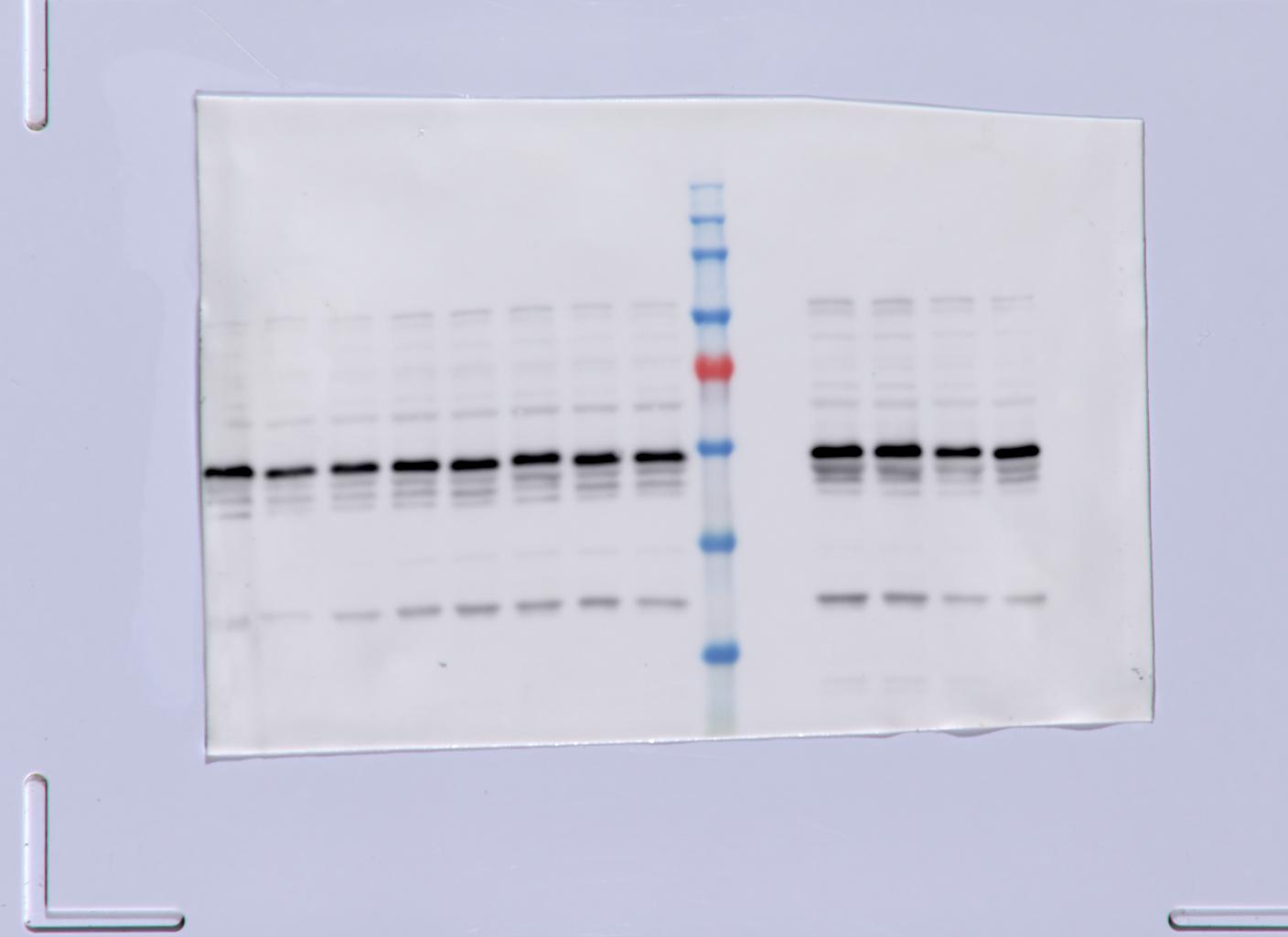

Supplement: Source data 1. [file elife-78197-data1.zip › ActC_Source_Files/ActC_SFig4_Topleft_Smad2.jpg]

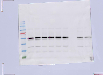

Supplement: Source data 1. [file elife-78197-data1.zip › ActC_Source_Files/ActC_SFig4_MiddleRight_Smad2.png]

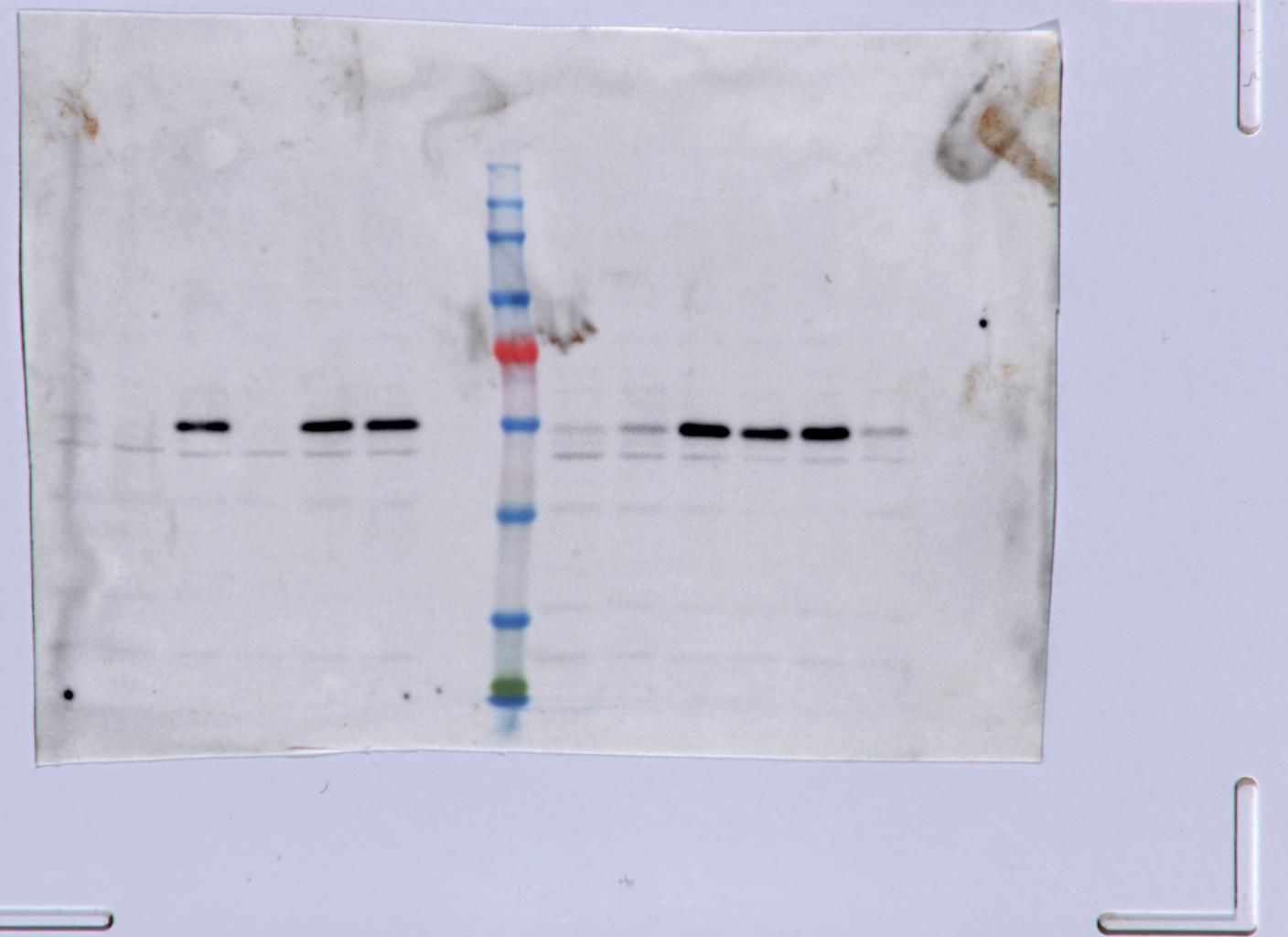

Supplement: Source data 1. [file elife-78197-data1.zip › ActC_Source_Files/ActC_SFig4_TopRight_pSmad2.jpg]

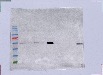

Supplement: Source data 1. [file elife-78197-data1.zip › ActC_Source_Files/ActC_SFig4_MiddleRight_pSmad2.jpg]

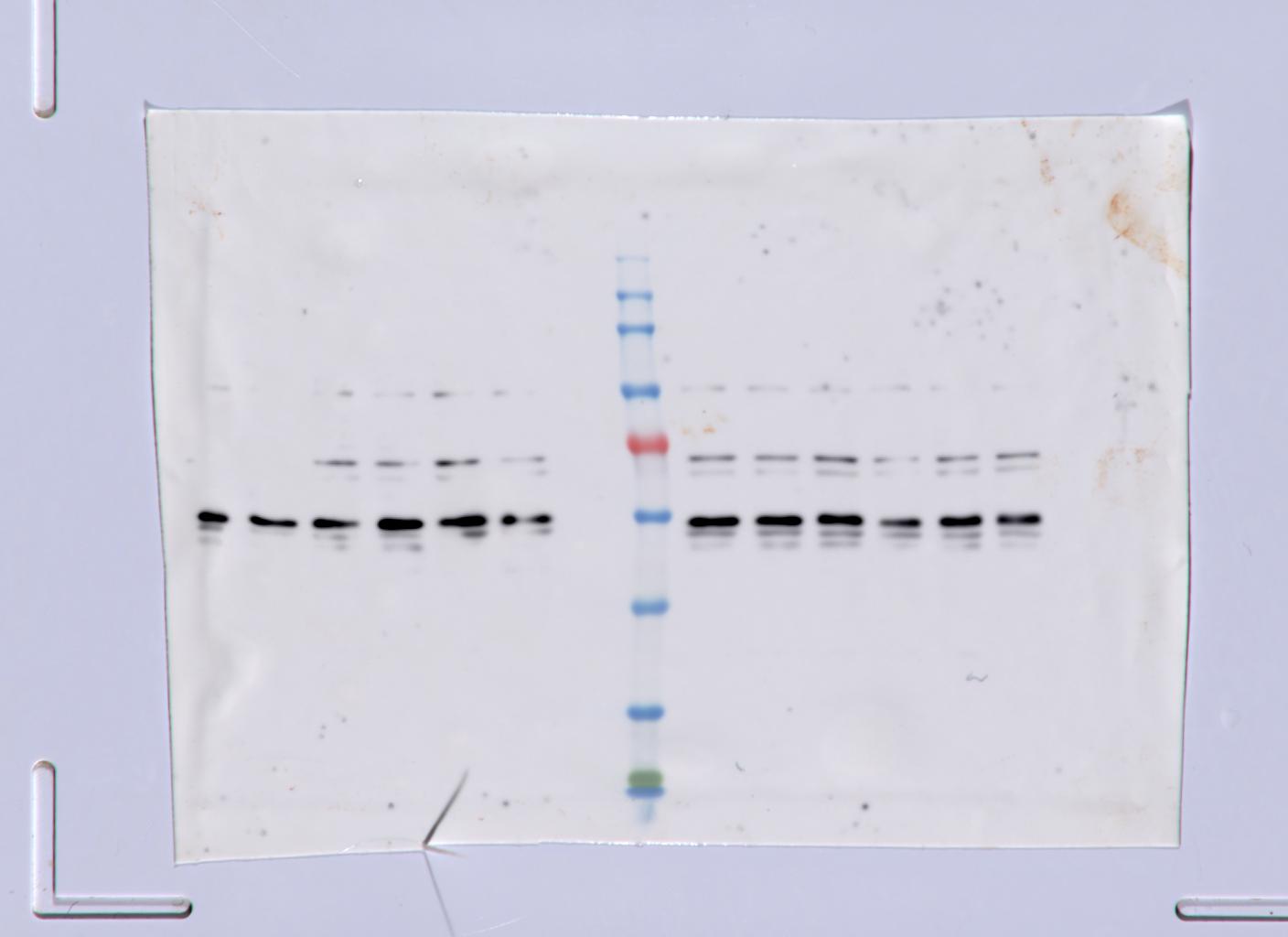

Supplement: Source data 1. [file elife-78197-data1.zip › ActC_Source_Files/ActC_SFig4_TopRight_Smad2.jpg]

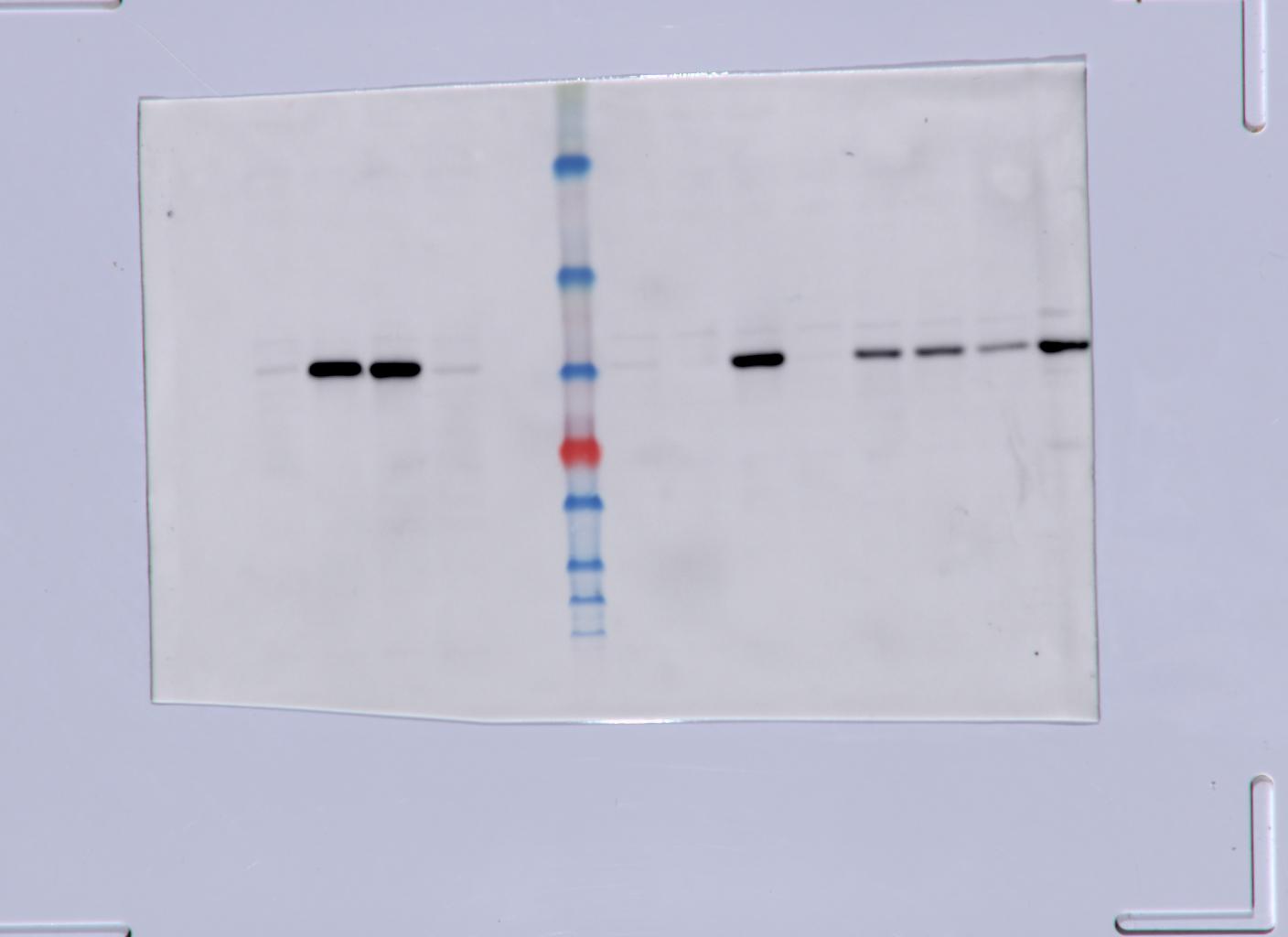

Supplement: Source data 1. [file elife-78197-data1.zip › ActC_Source_Files/ActC_SFig4_Topleft_pSmad2.jpg]

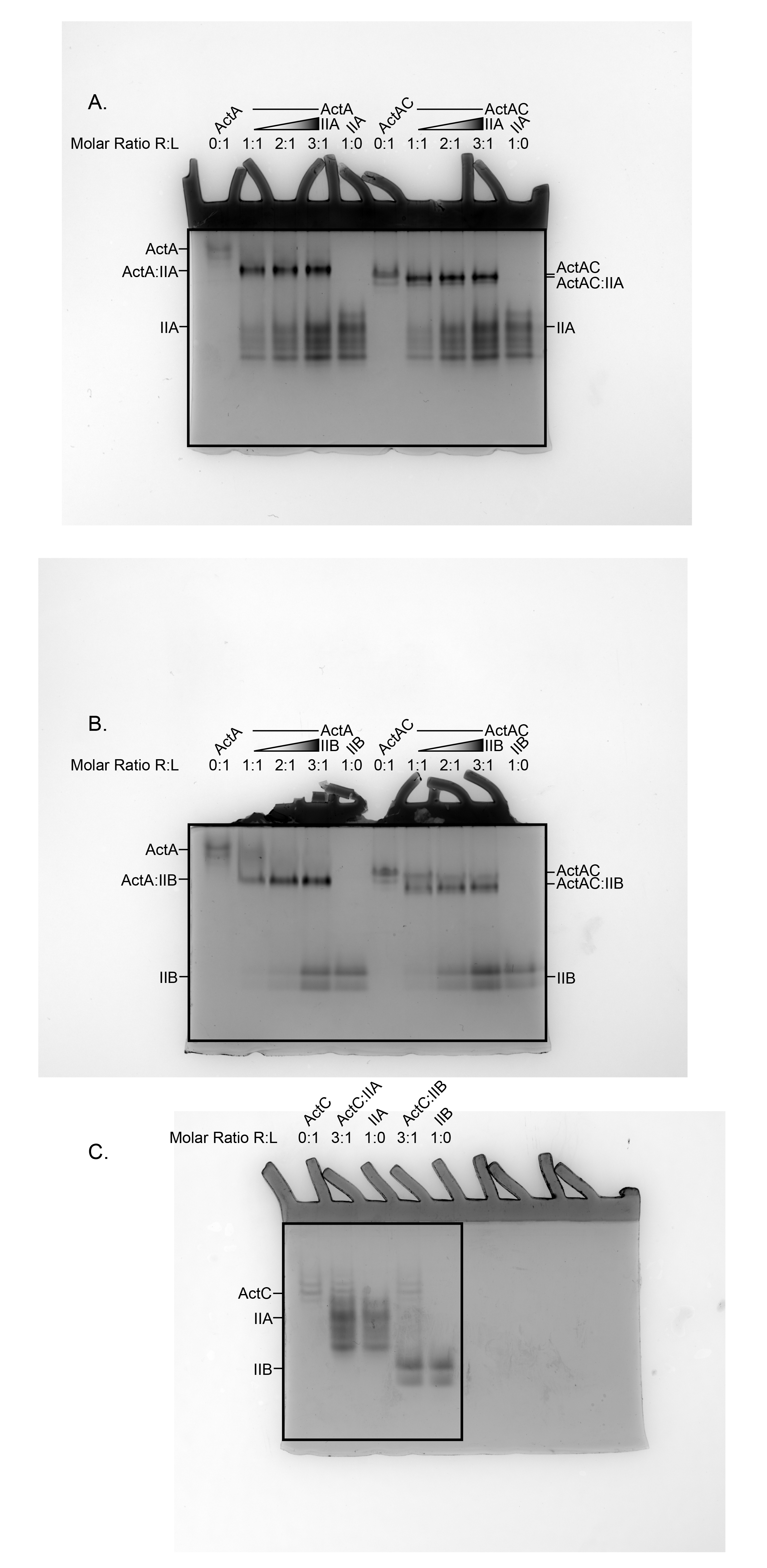

Supplement: Source data 1. [file elife-78197-data1.zip › ActC_Source_Files/ActC_SFig4_Raw_Gels_Labeled.png]

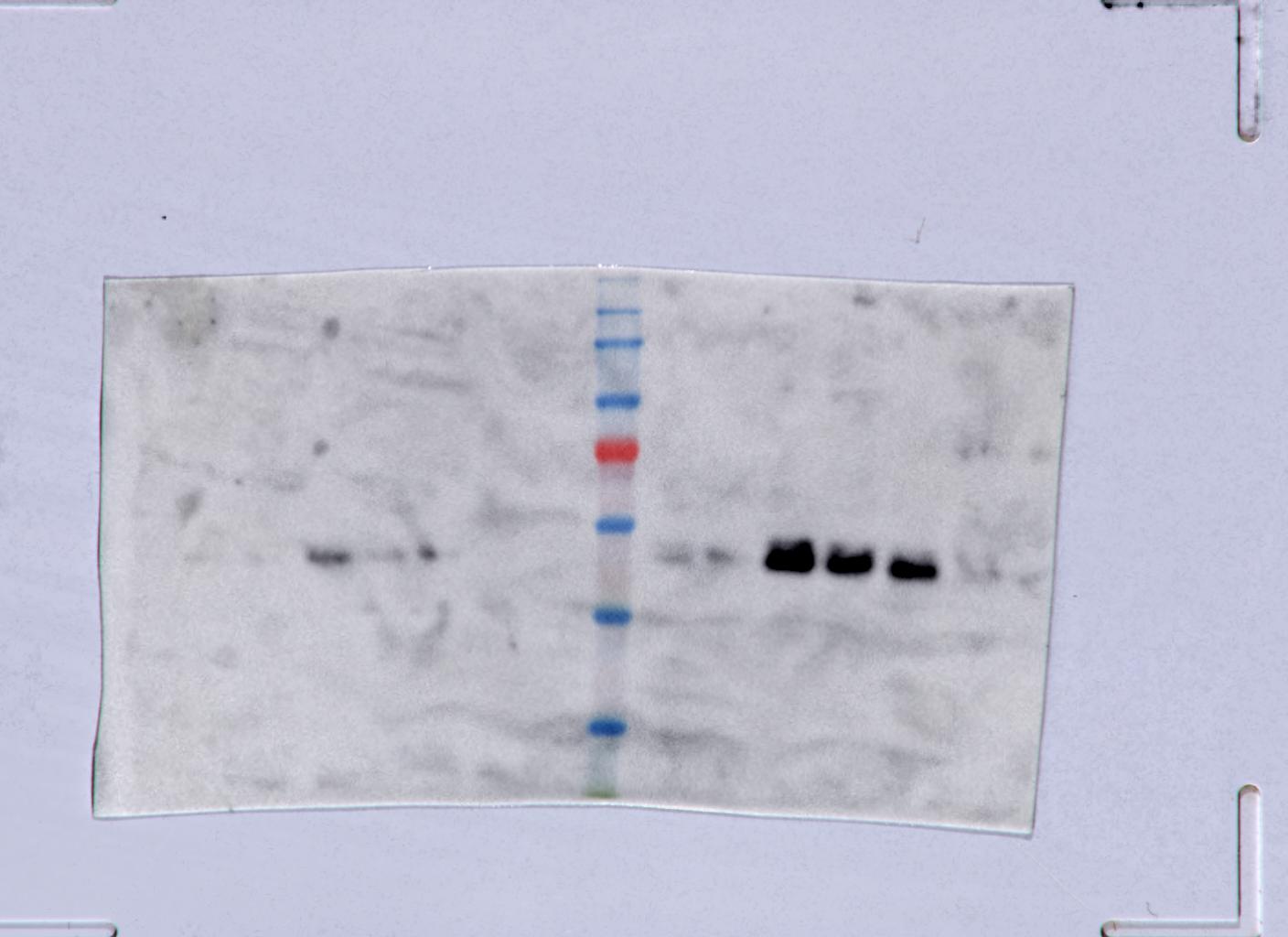

Supplement: Source data 1. [file elife-78197-data1.zip › ActC_Source_Files/ActC_SFig4_BottomMiddle_pSmad2.jpg]
